# Inhibition of Neuroblastoma Tumor Growth by Targeted Delivery of MicroRNA-34a Using Anti-Disialoganglioside GD_2_ Coated Nanoparticles

**DOI:** 10.1371/journal.pone.0038129

**Published:** 2012-05-25

**Authors:** Amanda Tivnan, Wayne Shannon Orr, Vladimir Gubala, Robert Nooney, David E. Williams, Colette McDonagh, Suzanne Prenter, Harry Harvey, Raquel Domingo-Fernández, Isabella M. Bray, Olga Piskareva, Catherine Y. Ng, Holger N. Lode, Andrew M. Davidoff, Raymond L. Stallings

**Affiliations:** 1 Department of Molecular and Cellular Therapeutics, Royal College of Surgeons in Ireland, Dublin, Ireland; 2 National Children’s Research Centre, Our Lady’s Children’s Hospital, Dublin, Ireland; 3 Department of Surgery, St. Jude Children’s Research Hospital, Memphis, Tennessee, United States of America; 4 Department of Surgery, University of Tennessee Health Science Center, Memphis, Tennessee, United States of America; 5 Biomedical Diagnostics Institute, Dublin City University, Dublin, Ireland; 6 Department of Paediatrics and Paediatric Haematology/Oncology, University of Greifswald, Greifswald, Germany; Institute of Cancer Research: Royal Cancer Hospital, United Kingdom

## Abstract

**Background:**

Neuroblastoma is one of the most challenging malignancies of childhood, being associated with the highest death rate in paediatric oncology, underlining the need for novel therapeutic approaches. Typically, patients with high risk disease undergo an initial remission in response to treatment, followed by disease recurrence that has become refractory to further treatment. Here, we demonstrate the first silica nanoparticle-based targeted delivery of a tumor suppressive, pro-apoptotic microRNA, miR-34a, to neuroblastoma tumors in a murine orthotopic xenograft model. These tumors express high levels of the cell surface antigen disialoganglioside GD2 (GD_2_), providing a target for tumor-specific delivery.

**Principal Findings:**

Nanoparticles encapsulating miR-34a and conjugated to a GD_2_ antibody facilitated tumor-specific delivery following systemic administration into tumor bearing mice, resulted in significantly decreased tumor growth, increased apoptosis and a reduction in vascularisation. We further demonstrate a novel, multi-step molecular mechanism by which miR-34a leads to increased levels of the tissue inhibitor metallopeptidase 2 precursor (TIMP2) protein, accounting for the highly reduced vascularisation noted in miR-34a-treated tumors.

**Significance:**

These novel findings highlight the potential of anti-GD_2_-nanoparticle-mediated targeted delivery of miR-34a for both the treatment of GD_2_-expressing tumors, and as a basic discovery tool for elucidating biological effects of novel miRNAs on tumor growth.

## Introduction

Neuroblastoma is a childhood cancer derived from precursor cells of the sympathetic nervous system, occurring primarily in children under the age of 5 years. Although neuroblastoma is associated with a high death rate in pediatric oncology, the disease is highly heterogeneous with respect to clinical behaviour, ranging from spontaneous regression to rapid progression [1]. Low risk neuroblastoma patients have a cure rate of greater than 90%, while high risk patients who are diagnosed over the age of 1 year have a poor prognosis, with approximately 40% survival rates. Tumors with amplification of the *MYCN* transcription factor or loss of heterozygosity for a large segment of chromosome 11 q represent two distinct genetic subtypes of the disease with particularly aggressive clinical phenotypes and poor patient survival. Patients with high risk neuroblastoma treated with intensive multi-modal chemotherapy often show an initial remission, however, disease recurrence that is refractory to further treatment is common.

GD_2_ is a glycolipid highly expressed on the cell surface of neuroblastoma and several other cancers [2], providing a potential target for immunotherapy and therapeutic targeting [3]–[6]. GD2 expression in neuroblastoma tumour samples is substantial [7] and uniform [8] and recent immunotherapy studies with a human/mouse chimeric disialoganglioside GD2 (GD_2_) antibody (ch14.18) combined with cytokine administration resulted in a significant improvement in neuroblastoma patient outcome [9] suggesting that the development of therapeutics directed to GD_2_ is a promising concept [10]–[12]. Although anti-GD2 immunotherapy was significantly superior to standard therapy for event-free survival (EFS) (66±5% vs. 46±5%) and overall survival (OS) (86±4% vs. 75±5%) at two years post-treatment [9], longer term survival rates have not yet been published. Anti-GD_2_ therapy was found to result in substantial pain and additional deleterious side-effects, potentially due to GD_2_ expression in peripheral neural tissue [9].

MicroRNAs (miRNAs) are small RNAs that regulate gene expression at a post-transcriptional level [13], [14]. Expression profiling studies of neuroblastoma primary tumors have identified many miRNAs whose expression levels have been significantly associated with poor patient survival [15]–[22], and functional studies have demonstrated that several miRNAs are capable of inducing apoptosis or differentiation when ectopically over-expressed in neuroblastoma cell lines [23]–[29]. The inhibition of endogenous oncogenic miRNAs by antagomirs; or replacement of tumor suppressive miRNAs, would represent a novel method of treating neuroblastoma. Although miRNA-mediated cancer therapeutics have been the subject of intensive research [21], [22], [30], the successful application of miRNAs as a cancer therapy *in vivo* is very limited [31]–[35].

A miRNA that targets multiple genetic pathways involved with cancer cell proliferation, apoptosis or differentiation would be most desirable for use as a potential therapeutic, as mutation of multiple target sites would be required for cancer cells to become resistant. The multi-gene targeting nature of miR-34a is well documented, with target transcripts including *MYCN, BCL2, SIRT1, NOTCH1, JAG1, CCND1, CDK6* and *E2F3* [23], [24], [28], [36]–[41]. MiR-34a induces activation of a caspase-mediated apoptotic pathway when over-expressed in neuroblastoma cell lines, making it ideally suited for miRNA-mediated therapeutics [23]. However, miRNA replacement therapy would require a method for stable and targeted delivery to tumors.

Here, we demonstrate for the first time the targeted delivery of miR-34a to neuroblastoma tumors using a disialoganglioside GD2 (GD_2_)-antibody conjugated to the surface of porous silica nanoparticles systemically administered to a well characterized murine orthotopic xenograft disease model [42]. Although use of GD_2_-Ab ch14.18 as a targeting antibody for liposome-mediated therapeutic delivery has previously been evaluated [43], [44], the main disadvantages of standard liposome formulations include lower drug-loading capacity and rapid drug leakage compared to the use of nanoparticles of identical size [45]. In this study, we evaluated silica-based nanoparticles [46] which are considered a more stable and inert delivery vehicle with biodegradable and non-toxic properties both *in vitro* and *in vivo* [47]–[49]. Analysis of tumors treated with miR-34a by immuohistochemical staining indicated that multiple mechanisms, including increased apoptosis, and surprisingly, decreased angiogenesis, were responsible for the anti-tumorigenic effects of this miRNA. In addition, we identify a novel multi-step molecular mechanism for the anti-angiogenic impact of miR-34a on neuroblastoma tumors.

## Results

### Specific Uptake of Anti-GD_2_ Conjugated Nanoparticles Containing miRNA and the Effect on Cell Growth in vitro

**Figure 1:**
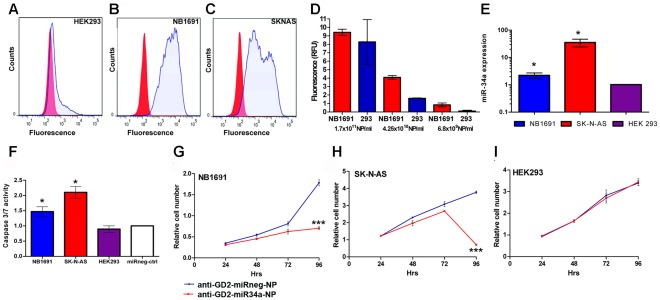
Specific uptake of anti-GD_2_-nanoparticles exclusively by GD_2_ expressing cells. Effects on growth and caspase activity by anti-GD_2_ conjugated nanoparticles containing miR-34a. (A) FACS analysis, using an anti-GD_2_ primary antibody and goat anti-mouse IgG2a-PE secondary antibody indicated little, if any, GD_2_ surface antigen on HEK293 cells. Conversely NB1691 (B) and SK-N-AS (C) cells showed significant GD_2_ reactivity. (D) Varying concentrations of FITC-anti-GD_2_-NPs were added to cell culture media of NB1691 or HEK293 (1×10^6^ cells). A concentration of 6.8×10^9^ particles/ml (40 µg/ml) was the optimal dosage tested *in vitro* for specific delivery of encapsulated FITC fluorophore to NB1691 neuroblastoma cells with minimal incorporation into HEK293 cells. (E) Anti-GD2-miR34a or control-anti-GD2-nanoparticles were added to adhered neuroblastoma SK-N-AS and NB1691, and to HEK293 cells for 4 hours in standard media. Media was removed, the cells washed and media was replaced. RNA was isolated from these cells after 24 hours and assessed for miR34a levels. Anti-GD_2_-miR34a-NPs treatment (40 µg/ml) led to ∼2 fold increase in miR-34a levels in NB1691 cells, ∼30 fold increase in SK-N-AS cells (*p<0.05, n=3), with no significant change in miR-34a transcript levels in HEK293 cells. (F) NB1691 and SK-N-AS cells showed a significant increase in caspase 3/7 activity relative to anti-GD_2_-miRneg-NP-treated cells after 72 hrs (*p<0.05, n=3) while HEK293 cells showed no significant increase in caspase activity under these conditions. Acid phosphatase assays indicated a significant reduction in viable cell numbers for (G) NB1691 and (H**)** SK-N-AS cells treated with anti-GD_2_-miR34a-NPs, over a 96 hour period (***p<0.001) relative to anti-GD_2_-miRneg-NP-treated controls. (I) HEK293 cells showed no significant reduction of viable cells following treatment with anti-GD_2_-miR34a-NP (p>0.05, n=3).

The use of anti-GD_2_ conjugated silica nanoparticles as a stable system for targeted delivery of miR-34a was extensively evaluated *in vitro* prior to use in a published murine orthotopic xenograft model of neuroblastoma [42]. As illustrated in Figure S1, the silica network of the nanoparticles is dissolved by a hydrolysis dependent process. The *in vivo* model utilizes two cell lines which were modified to stably express luciferase, NB1691 (MYCN amplified) and SK-N-AS (derived from a non-MYCN amplified tumor with LOH for chromosome 11 q). Both of these cell lines, along with a negative control cell line, HEK293 (embryonic kidney origin), were initially assessed by fluorescence activated cell sorting (FACS) for GD_2_ surface protein expression. FACS analysis indicated that GD_2_ expression was significantly higher in the two neuroblastoma cell lines relative to HEK293 (Figure 1A–C). In order to determine if anti-GD_2_ conjugated nanoparticles would then bind and be internalised by GD_2_ expressing cells at greater specificity than GD_2_ negative cells, NB1691 and HEK293 were treated with different concentrations of anti-GD_2_ conjugated nanoparticles doped with FITC (Figure 1D). Four hours after treatment, cells were washed to remove non-internalised nanoparticles, lysed and FITC fluorescence, as a result of nanoparticle uptake and intercellular degradation, was measured using a luminometer. A final concentration of 6.8×10^9^ particles/ml (40 µg/ml) yielded uptake and subsequent FITC release that was approximately 7.6 fold higher in NB1691 cells relative to HEK293 (normalised to non-coated FITC-doped nanoparticles). Notably, higher concentrations of GD_2_-FITC-NPs resulted in some nanoparticle uptake by HEK293 cells, consistent with the results of FACS analysis indicating a minimal level of GD_2_ expression on the cell surface (Figure 1A).

In order to evaluate if miRNAs which are non-covalently encapsulated within the silica matrix of the nanoparticle would be released into the cellular cytoplasm after uptake, we constructed anti-GD_2_-conjugated nanoparticles encapsulating either miR-34a or a scrambled negative oligonucleotide. Treatment of NB1691, SK-N-AS and HEK293 cells *in vitro* with 6.8×10^9^ particles/ml (40 µg/ml) resulted in a significant up-regulation of miR-34a in the neuroblastoma cell lines, but not in HEK293 (Figure 1E), indicating exclusive binding and release of the nanoparticle contents to GD_2_ expressing cells. Moreover, the release of miR-34a in NB1691 and SK-N-AS cells resulted in a significant increase in caspase 3/7 activation (Figure 1F *p<0.05) and a decrease in cell viability, as determined by both an acid phosphatase assay (Figure 1G–H ***p<0.001) and cell counting (Figure S2). No apoptotic response was detected for HEK293 cells, due to the specificity of the anti-GD_2_-NPs for GD_2_ expressing cells (Figure 1I). To confirm that HEK293 cells are susceptible to miR-34a induced apoptosis, miR-34a was transfected into these cells, resulting in ectopic over-expression (Figure. S3A) and a significant decline in cell viability (Figure S3B). We also determined that other cell lines of non-malignant origin, such as IMR-90 fibroblasts are also adversely affected by miR-34a (Figure S4A and B), further underscoring the importance for the development of a system for targeted delivery *in vivo*.

### Targeting of Neuroblastoma Tumors by Systemically Administered Anti-GD_2_ Conjugated Nanoparticles

FITC-anti-GD_2_-NPs or FITC-NPs were systemically administered to tumor bearing mice at day 14, after tumor establishment had been verified.

**Figure 2:**
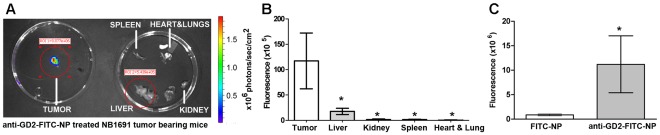
Targeting of neuroblastoma by systemically administered anti-GD_2_ conjugated nanoparticles. (A) Systemic administration through lateral tail injection of FITC-anti-GD_2_-NPs (1 mg/ml) resulted in targeted delivery of FITC dye predominantly to tumors (indicated by fluorescence intensity color map generated from scans using IVIS instrumentation). (B) FITC-anti-GD_2_-NP-treated organs have significantly less fluorescence than isolated tumors (*p<0.05, n=4−5) and **(C)** FITC-anti-GD_2_-NP treated tumors showed significantly greater presence of FITC dye relative to FITC-NP treated tumors (*p<0.05, n=4−5).

The mean fluorescence intensities of tumors were 6.7 to 258 fold greater than in healthy organs (liver, spleen, kidney, lung and heart) scanned *ex vivo* (Figure 2A,B), indicating the specificity of anti-GD_2_-NPs. Among the normal organs, FITC signal was highest in liver, which could potentially be due to the detoxification function of this organ. Most importantly, mean fluorescence intensity of tumors from mice injected with FITC-anti-GD_2_-NPs was 13.9 fold higher (Figure 2C ; *p<0.05) than tumors from mice injected with FITC-nanoparticles lacking GD_2_ antibody, confirming targeted delivery (Figure 2C).

### Anti-tumorigenic Effect of Anti-GD_2_ Conjugated Nanoparticles Bearing miR-34a in vivo

**Figure 3:**
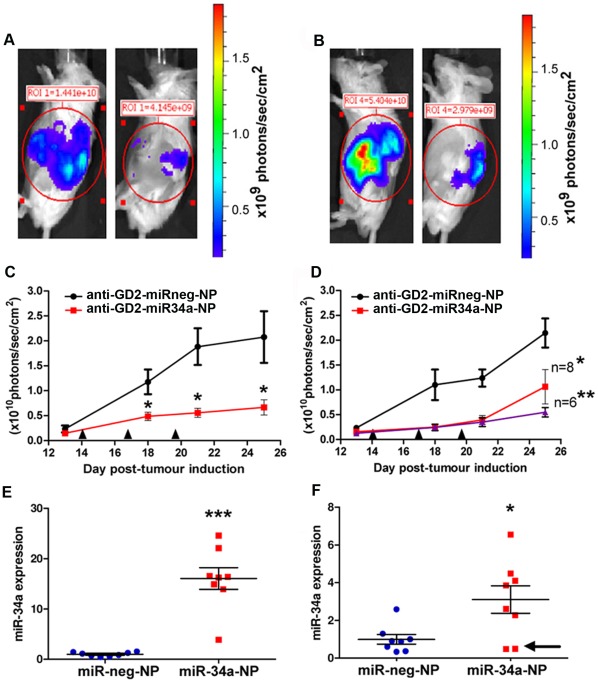
Anti-neuroblastoma effect of anti-GD_2_ conjugated nanoparticles bearing miR-34a in vivo. Bioluminescent images representative of mice bearing (A) NB1691^luc^ tumors treated with anti-GD_2_-miRneg-NP (left) or anti-GD_2_-miR34a-NP (right), along with representative mice bearing (B) SK-N-AS^luc^ tumors treated with anti-GD_2_-miRneg-NP (left) or anti-GD_2_-miR34a-NP (right) were obtained at day 25. Tumor growth curves from (C) mice bearing NB1691^luc^ tumors treated with anti-GD_2_-miRneg-NP (black line) or anti-GD_2_-miR34a-NP (red line) and (D) mice bearing SK-N-AS^luc^ tumors treated with anti-GD_2_-miRneg-NP (black line) or anti-GD_2_-miR34a-NP (red line). Time points for systemic administration of nanoparticles are indicated by the symbol ▴. Differences in tumor growth between mice injected with anti-GD_2_-miR34a-NP versus anti-GD_2_-miRneg-NP were statistically significant for both models (NB1691^luc^ n=8, *p<0.05) (SK-N-AS^luc^ *p<0.05 for n=8 and **p<0.01 for n=6). For the SK-N-AS^luc^ model, two tumors grew significantly faster than the median and are represented in the growth curve for all 8 tumors (red line). The purple line represents the growth curve without these two tumors (n=6). Mature miR-34a transcript levels were significantly higher in anti-GD_2_-miR34a-NP treated tumours relative to anti-GD_2_-miRneg-NP-treated control tumors in both the (E) NB1691^luc^ (***p<0.001, n=8) and (F) SK-N-AS^luc^ (*p<0.05, n=8). Notably, the two SK-N-AS tumors with increased growth from (D) had poor uptake of miR-34a (arrow).

Given the success of our *in vivo* targeting experiments, we then evaluated anti-GD_2_-coated nanoparticles encapsulating miR-34a to assess the impact on neuroblastoma tumors using the NB1691^luc^ and SK-N-AS^luc^ orthotopic xenograft models. Cohorts of NB1691^luc^ or SK-N-AS^luc^ tumor bearing mice were systemically administered either anti-GD_2_-miR-34a-NP or anti-GD_2_-miRneg-NP (containing a scrambled oligonucleotide) at day 14, 17 and 20 post-tumor establishment. Equivalency of tumor sizes in miR-34a versus negative control treated populations was confirmed by ultra-sonography and bioluminescence analysis prior to injection (at day 13 post-tumor induction; Figure S5). Monitoring of tumors by bioluminescent imaging on days 18, 21 and 25 revealed a significant reduction in the bioluminescent intensity of NB1691^luc^ and SK-N-AS^luc^ tumors treated with anti-GD_2_-miR-34a-NPs relative to negative control (Figure 3A–D). Measurements of post-mortem tumor volumes and weights (day 25) confirmed the highly significant reduction in tumor growth in anti-GD_2_-miR34a-NP treated mice (Figure S6). Significantly enhanced miR-34a levels were detected by qPCR in NB1691^luc^ and SK-N-AS^luc^ tumors treated with anti-GD_2_-miR34a-NP relative to those treated with anti-GD_2_-miRneg-NP (Figure 3E and F). Interestingly, two of the SK-N-AS^luc^ tumors which grew faster than the median had no increase in miR-34a levels (Figure 3D and F), indicating that they had not been successfully or significantly transduced by anti-GD_2_-miR34a-NPs.

Evaluation of non-specific delivery of miR-34a to normal organs was paramount to ensuring specific targeting of the anti-GD_2_-NPs to the neuroblastoma tumor. MiR-34a levels in healthy liver, kidney and lung from each animal treated with either anti-GD_2_-miR34a-NP or anti-GD_2_-miRneg-NP were evaluated by qPCR to assess potential off-target delivery of the miRNA. None of the organs exhibited any significant increase in mature miR-34a transcript levels subsequent to treatment, relative to anti-GD_2_-miRneg-NP treated controls (Figure S7). Blood chemistries were analysed in anti-GD_2_-miRneg-NP and anti-GD_2_-miR34a-NP-treated animals to evaluate the effects, if any, administration of silica nanoparticles might have on the animal. From the data analysed (Figure S8), systemic tail vein injection of either miR-negative control or miR-34a– anti-GD_2_-nanoparticles did not appear to cause significant adverse effects on kidney and/or liver function. It is also noteworthy that mice treated with anti-GD_2_-miR34a-NP (three doses of 1.7×10^11^ NP/ml [1 mg/ml]) appeared active and healthy throughout the course of the treatment period, devoid of symptoms such as neuropathic pain which has been associated with anti-GD_2_ immunotherapy in patients [50]. The equivalent total amount of anti-GD2 attached to the surface of the nanoparticles injected into mice was ∼3.6 fold less than what is used for immunotherapy in humans, which could explain why the mice had no detectable side effects associated with pain. This calculation is based on a total dose in humans of 56.76 mg antibody/kg body weight, administered in 6 cycles over 14 days.

### miR-34a Over-expression Launches a Cascade of Molecular Events in Tumors

**Figure 4:**
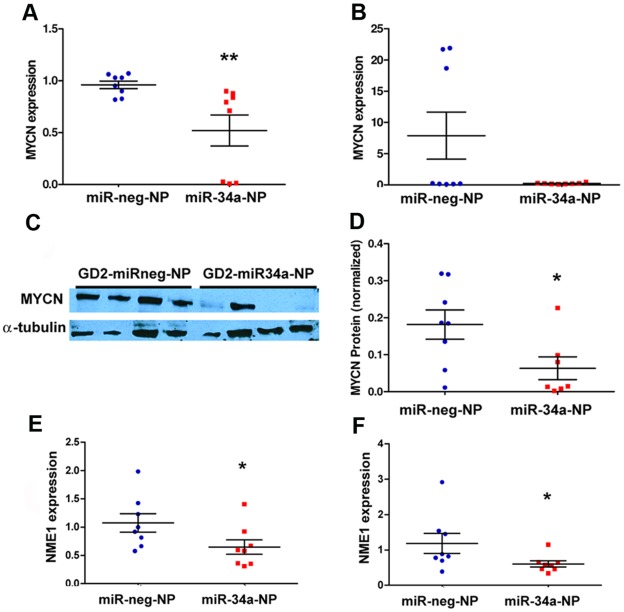
Targeting of MYCN by miR-34a in vivo. (A) MYCN, a validated target of miR-34a, mRNA levels were significantly reduced in GD_2_-miR34a-NP treated NB1691^luc^ tumors relative to negative controls (**p<0.001, n=8) (B) qPCR analysis of SK-N-AS^luc^ tumors did not indicate a statistically significant difference between anti-GD_2_-miR34a-NP and anti-GD_2_-miRneg-NP treated tumours, potentially because levels of MYCN expression are very low in this cell line. Three SK-N-AS tumors treated with negative control nanoparticles appeared to have somewhat higher expression then the median, although this level of expression is very low compared to MYCN amplified tumors (C) MYCN reduction in NB1691^luc^ was validated at a protein level by Western Blot and (D) protein suppression was quantified using densitometry. *NME1*, a validated MYCN target, had reduced mRNA levels in anti-GD_2_-miR34a-NP treated NB1691^luc^ tumors (E) and SK-N-AS^luc^ tumors (F) relative to negative controls (*p<0.05, n=8), as evaluated by qPCR.

The levels of *MYCN*, a validated target of miR-34a that is amplified in some neuroblastoma tumors and cell lines, were assessed to further understand the molecular mechanisms leading to a reduction in tumor growth. The results of qPCR analysis highlighted a significant decrease in *MYCN* mRNA levels in the NB1691^luc^ tumors (*MYCN* amplified) (Figure 4A). There was not a statistically significant difference between anti-GD_2_-miR34a-NP relative anti-GD_2_-miRneg-NP. This is not surprising given the very low level of MYCN expression in SK-N-AS (Figure 4B). The decrease in *MYCN* mRNA in NB1691^luc^ tumors was validated at protein level by western blot (Figure 4C and D). *NME1*, which is transcriptionally activated by MYCN [51], was down-regulated in the tumors treated with anti-GD_2_-miR34a-NP relative to anti-GD_2_-miRneg-NP, illustrating that miR-34a was also producing secondary effects in addition to its direct targeting of 3′ UTRs (Figure 4E and F).

**Figure 5:**
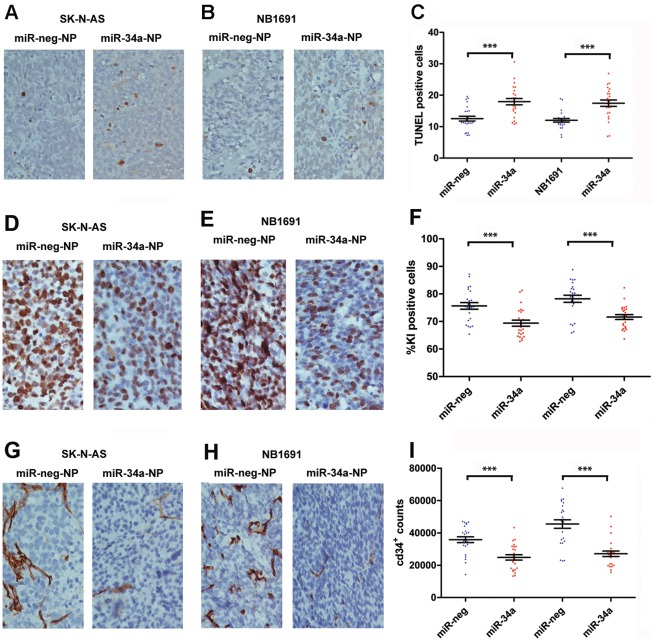
Pro-apoptotic and anti-angiogenic activity of GD_2_ targeted nanoparticles containing miR-34a. Tumors of mice treated with anti-GD_2_-miR-34a-NPs and negative controls were analyzed by TUNEL staining on paraffin embedded section of SK-N-AS^luc^ (a) and NB1691^luc^ (b) tumors. In both tumor subtypes, treatment with anti-GD_2_-miR34a-NPs led to a significant increase in apoptosis (**c**, ***p<0.001, n=8). Immunohistochemistry staining with CD34 showed a marked decrease in the endothelial cell marker subsequent to anti-GD_2_-miR34a-NP treatment (d-f, ***p<0.001, n=8). KI67 staining showed reduced proliferation in anti-GD_2_-miR34a-NP treated cohorts (g-i**,** ***p<0.001, n=8).

Targeted delivery and over-expression of miR-34a resulted in a significant increase in apoptosis, as demonstrated by TUNEL staining of paraffin embedded tumor sections of both NB1691^luc^ and SK-N-AS^luc^ tumors (Figure 5A–C). Cell proliferation was also significantly reduced in the miR-34a treated tumors, as determined by KI67 immunohistochemical staining (Figure 5D–F). CD34 immunohistochemical staining, which detects vascular endothelial cells, indicated that over-expression of miR-34a also had a very significant negative impact on tumor vascularisation, or angiogenesis (Figure 5G–I).

### Molecular Events Associated with Decreased Tumor Vascularisation

**Figure 6:**
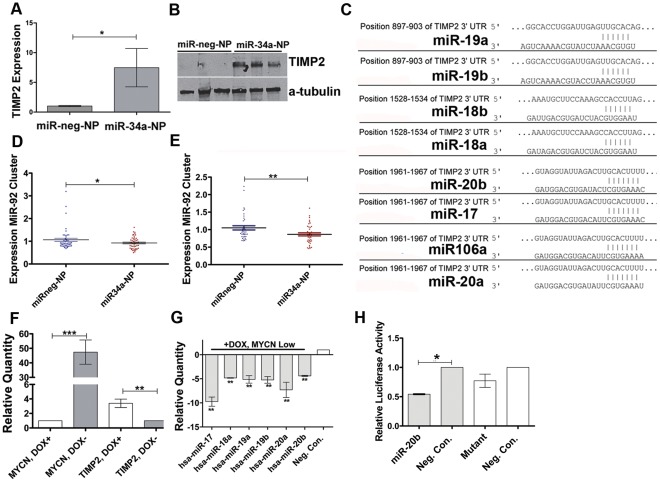
Molecular events involved with miR-34a anti-angiogenic effects. (A) qPCR and (B) western blot analyses of TIMP2 mRNA (n=4 tumors) and protein levels (n=3 tumors) from NB1691 tumors treated with anti-GD-miR-neg-NP or anti-GD20miR34a-NP. (C) Computationally predicted target sites on the 3′ UTR of *TIMP2* for miR-92 polycistronic cluster members from chromosome 13 and X. qPCR analysis of miR-92 cluster members from chromosome 13 (D) and chromosome X (E) in tumors treated with anti-GD-miR-neg-NP (n=8) or anti-GD20miR34a-NP (n=8). (F) qPCR analysis of MYCN and TIMP2 expression in SHEP TET21 cells possessing a repressible MYCN transgene. Expression levels of these genes in cells treated (DOX+) and untreated (DOX-) with doxycycline are displayed. (G) Expression levels of miR-92 cluster members in SHEP TET21 following treatment with doxycycline (MYCN low expression state)(n=5 biological replicate experiments). (H) Luciferase assays following co-transfection of NB1691 cells with a reporter construct containing a wild type *TIMP2* 3′ UTR region and either miR-20b mimics or a negative control oligonucleotide (n=2). Luciferase activity following co-transfection of the same cell line with a plasmid having a mutated binding site for miR-20b and either miR-20b mimics or a negative control oligonucleotide is also displayed.

Although the positive impact of miR-34a on promoting neuroblastoma cell apoptosis is well documented [23], the mechanism leading to the observed decrease in angiogenesis is poorly understood. To further elucidate this mechanism, mRNA from four NB1691 tumors treated with anti-GD_2_-miR34a-NP, along with an equivalent number of negative control tumors, were analyzed using TaqMan low density arrays representing 95 genes involved with angiogenesis. Of note, mRNA levels for tissue inhibitor of metalloproteinases-2 (*TIMP2*) were significantly up-regulated in the miR-34a treated tumors relative to the negative controls (Figure 6A) and this increase was also validated at protein level (Figure 6B). This finding is highly significant given that TIMP2 is an important anti-angiogenic factor [52], [53].

To explore the possibility that MYCN might be directly binding to the TIMP2 promoter and directly repressing transcription, we examined previously published genome-wide MYCN binding site data for neuroblastoma [54]. There was no enrichment for MYCN binding at this promoter. We also assessed the possibility that the up-regulation of *TIMP2* might be mediated by a targeting miRNA that had become down-regulated in the tumors treated with anti-GD_2_-miR34a-NP. Intriguingly, several members of the oncogenic miR-92 polycistronic clusters mapping to chromosomes 13 and X, which are positively regulated by MYCN binding to their promoter regions [55], [56], are computationally predicted to target the *TIMP2* 3′ UTR (Figure 6C). Overall, members of the miR-92 clusters mapping to chromosome 13 and X had significantly lower expression in the miR-34a treated tumors relative to negative control, presumably due to the substantial decrease in MYCN levels resulting from miR-34a targeting (Figure 6D and E). In order to experimentally confirm that MYCN was repressing *TIMP2* through the up-regulation of miR-92 cluster members, we used the well characterized SHEP-TET21N cell line containing a repressible MYCN transgene to determine if TIMP2 levels increased when MYCN levels were repressed. Treatment of these cells with doxycycline repressed both MYCN expression (∼50 fold; Figure 6F) and the expression of miR-92 cluster members (5 to 10 fold; Figure 6G), resulting in an approximate 4 fold increase in *TIMP2* mRNA (Figure 6F).

It was also of interest that the expression of several miR-92 cluster members (17, 18a, 19a, 20a, 20b and 106a) was inversely correlated with *TIMP2* expression in a panel of primary neuroblastoma tumors, and that low expression of *TIMP2* in tumors is associated with poor overall and event free patient survival (Figure S9). In order to confirm that *TIMP2* was a direct target of miR-92 cluster members, a segment of the *TIMP2* 3′ UTR containing the miR-20b, miR-17-5p, miR-106a and miR-20a seed site (all the same sequence, Figure 6C) was cloned into a luciferase reporter plasmid. Co-transfection of this construct with miR-20b mimics into NB1691 cells significantly reduced luciferase activity relative to a negative control oligonucleotide (Figure 6H), while mutation of the seed site abrogated the effect of miR-20b on luciferase activity, thus we conclude that miR-20b, and potentially other miR-92 family members directly target *TIMP2*.

## Discussion

The targeted delivery of therapeutics to tumors is still a major challenge in cancer research, holding the potential to mitigate deleterious side effects, as well as increase the efficacy of treatment by delivering a higher concentration of drug or nucleic acid to the site of disease. To date, relatively few strategies have been developed to target silica nanoparticles to tumor cells using *in vivo* models. Patil *et al* [57] used biotin as a targeting ligand for delivery of nanoparticles to breast cancer xenograft tumors which over-express biotin receptors, demonstrating that targeted delivery permitted a greater accumulation of nanoparticles in the tumors. The tumor targeting antibody GC4 has also been used to successfully target silica nanoparticles containing siRNA and miRNA to melanoma cells in a murine model of melanoma metastasis [58]. In this study, we demonstrate that the bioconjugation of GD_2_ ch14.18 antibody to the surface of porous silica nanoparticles containing miR-34a results in the targeted delivery of these functionalized nanoparticles and significant inhibition of neuroblastoma tumor growth in a murine orthotopic disease model. The use of GD_2_ antibody coated silica nanoparticles as a delivery vehicle might also be applicable to other tumors of neuroectodermal origin, such as melanoma, which express high levels of the surface antigen GD_2_ [59]. In addition to being a potential therapeutic, the use of GD_2_ antibody conjugated nanoparticles for targeted delivery of miRNAs also represents a basic discovery tool for elucidating the effects of novel miRNAs on tumor growth.

The GD_2_ antibody acts as a docking mechanism to the corresponding GD_2_ antigen, leading to receptor-mediated endocytosis via clathrin-coated pits and formation of endosomal compartments within the cell [60], [61]. The precise mechanism of nanoparticle-cargo release has not fully been elucidated; however, endosomal acidification might play a role in nanoparticle degradation and content release. Degradation and dye content release of synthesized nanoparticles used during this study was confirmed *in situ* (Figure S1) proving the degradative properties of these delivery vehicles. The use of GD_2_ antibody in the treatment of cancers leads to antibody dependent cellular cytotoxicity (ADCC) [10], [11], complement dependent cytotoxicity (CDC) [62], induction of the anti-idiotypic network [63] and apoptosis [64]. It is unlikely that the presence of GD_2_ antibody conjugated to the surface of the nanoparticle is contributing an anti-tumorigenic effect in this study, given that negative control particle also possessed conjugated GD_2_ antibody; and tumor progression within this cohort was extremely aggressive.

As demonstrated in this report, we were able to over-express miR-34a from 4 to 25 fold in NB1691 tumors, and 2 to 7 fold in SK-N-AS tumors. The lower incidence of uptake of miR-34a by SK-N-AS cells might be due to lower expression of GD_2_ on the cell surface. In this regard, it is interesting to note that FACs analysis for GD_2_ expression indicated a bimodal distribution for SK-N-AS, with a large population of cells clearly having less GD_2_ than NB1691 cells. Nevertheless, even a 2 to 7 fold increase in miR-34a levels negatively impacted SK-N-AS tumor growth.

Based on prior *in vitro* studies of miR-34a in neuroblastoma cell lines, we expected that targeted delivery of miR-34a to tumors in an orthotopic model would induce apoptosis [23]–[25], [28]. Although a statistically significant increase in apoptosis was detected, the over-expression of miR-34a in the tumors also had a profound negative impact on vascularization, as determined by CD34 staining. For several reasons, the inhibition of vascularisation is likely related to the decrease in MYCN activity caused by direct miR-34a targeting of its 3′ UTR. As demonstrated in this report, the decline in MYCN corresponded with a decrease in the levels of miR-92 polycistronic cluster members in NB1691 tumors, which are positively regulated by this transcription factor. TIMP2 is a direct target of some of these miRNAs, and therefore their down-regulation released TIMP2 from repression. TIMP2 inhibits angiogenesis through matrix metalloproteinases which are required to degrade the extracellular matrix and also through suppression of endothelial cell proliferation [52], [53]. However, the decline in MYCN mediated by miR-34a might also inhibit angiogenesis in other ways from those detailed in this publication. For example, Kang et al [65] demonstrated that siRNA mediated inhibition of *MYCN* leads to blocked secretion of vascular endothelial growth factor and reduced angiogenesis. More recently, Patterson *et al* [66] demonstrated that treatment of neuroblastoma cells with the MDM2 small molecule inhibitor, Nutlin-3a, significantly reduces angiogenesis, which is also consistent with our findings given that MYCN is a positive transcriptional regulator of *MDM2*. Thus, miR-34a up-regulation in tumors has an anti-angiogenic effect potentially mediated through direct inhibition of *MYCN*.

MiR-34a mediated inhibition of *MYCN* also holds potential benefits in the treatment of drug-resistant tumors given that MYCN has a documented role in the development of drug resistance. In neuroblastoma, direct regulation of ATP-binding cassette (ABC) transporters by MYCN implicates MYCN as a mediator of drug-resistance in neuroblastoma [67]. SiRNA-mediated MYCN suppression led to a down-regulation of the multidrug-resistance associated protein (MRP) gene and a corresponding increased sensitivity to a broad range of drugs including vincristine, doxorubicin, and sodium arsenate [68]. Moreover, miR-34a has been directly implicated in mediating chemo-drug resistance or sensitivity in a number of different cancers [69], [70].

Given that miR-34a has pro-apoptotic effects on cell lines of non-malignant origin, it seems likely that off-target delivery of this miRNA could have adverse effects on healthy vasculature and tissues. Targeted delivery could be particularly important in the context of paediatric malignancies, as off target delivery could potentially retard normal growth and development in young children. Although non-targeted lipid based delivery of miR-34a was shown to have anti-tumorigenic effects on a mouse xenograft model of prostate cancer, with the miR-34a formulation being tolerated by mice [33], targeting of miRNAs in humans, particularly paediatric patients, would be of particular importance in avoiding or limiting, long term deleterious and developmental effects such as those which currently occur with chemotherapeutic regimes as a result of off-target genetic alterations. Further studies in pre-clinical models to assess the impact, if any, of off-target delivery of miR-34a, would be of benefit to assess potential health risks.

Targeted delivery should provide a higher concentration of miR-34a to the tumor site, potentially increasing the efficacy of this mode of treatment. Future studies will be focused on determining optimal treatment doses and schedules, as well as optimizing encapsulation of chemotherapies in addition to miR-34a in porous silica nanoparticles for *in vivo* analysis of neuroblastoma.

## Materials and Methods

### Primary Neuroblastoma Tumours

Primary neuroblastoma tumours used in the Affymetrix Array microarray profiling were a subset of tumors previously described by Bray et al [15]. Twelve of the tumours were from a Tumor Bank at Our Lady’s Children’s Hospital, Dublin, and 30 came from the COG Tumor Bank, Phildelphia, P.A. The biological and clinical characteristics of the tumors are described in Table S1. The research was approved by the Ethics Committees of the Royal College of Surgeons in Ireland and Our Lady’s Children’s Hospital, Dublin, Ireland.

### Cell Lines

NB1691^luc^ and SK-N-AS^luc^ were engineered to express the enzyme firefly luciferase by the laboratory of Dr. Andrew Davidoff, as previously described [42], and maintained in RPMI-1640 supplemented with heat-inactivated foetal bovine serum (10%), l-glutamine (1%) and 100 µg/mL Zeocin (InVivoGen, San Diego, California). HEK293 cells were purchased from the European Collection of Cell Cultures and grown in DMEM supplemented with FBS (10%) and l-glutamine (1%). Each cell line was validated by aCGH profiling for identification of previously published genomic abnormalities.

### Reverse Transcription, Real-time qPCR and Western Blotting

Total RNA was extracted using a miRNeasy© kit (Qiagen Inc, Valencia, CA). Reverse transcription was carried out using 50 ng of total RNA and the TaqMan reverse transcription kit (Applied Biosystems). qPCR was carried out on the 7900 HT Fast Real-time System (Applied Biosystems). TaqMan qPCR probes and stem loop primers for miR-34a and miR-92 family members were obtained from Applied Biosystems. These probes only recognize mature miRNA sequences. β-actin, was used for normalization. A relative fold change in expression of target miRNA/gene transcripts was determined using the comparative cycle threshold method (2^−ΔΔCT^). Total protein was isolated from cells using a T-PER tissue protein extraction reagent (Thermo Scientific). Anti-MYCN antibody (n-mycn: Santa Cruz (SC-53993), 1∶500) or anti α-tubulin (Abcam (AB7291), 1∶5000 used for loading controls).

### Fluorescence Activated Sorting Analysis

Log phase cells (2×10^6^) were harvested and stained with GD_2_ primary antibody (Santa Cruz, sc-53831) and a goat anti-mouse IgG2a-PE secondary antibody (Santa Cruz, sc-3738). Cells were then analyzed by a FACS Calibur (BD Bioscience).

### Synthesis of FITC Dye Doped Silica Nanoparticle; Empty or Containing Either miR-34a or a miR-negative Control

All chemicals were obtained from Sigma Aldrich, (Sigma-Aldrich Corp., St. Louis, MO) unless otherwise stated. Silica nanoparticles (φ=74 nm) containing fluorescein (3 (w/w)) and synthetic premiR-34a (0.1 (w/w)), or a scrambled miR-negative control (0.1 (w/w)) were prepared using a microemulsion method [71]. The mechanism for formation of silica nanoparticles, outlined in Figure S1C, involves hydrolysis and condensation of a silica precursor (tetraethylorthosilicate). The proposed mechanism of drug delivery is dissolution of the nanoparticle and release of the dye under physiological conditions via hydrolysis of the silica network. This hydrolysis is simply the reverse of the condensation step which is also described in detail by Park *et al.* [72].

### Conjugation of GD_2_-Ab ch14.18 to the Nanoparticles

Conjugation was performed according to a previously published protocol using PAMAM dendrimers [73]. The NP–antibody bioconjugates were aliquoted (1.7×10^11^ nanoparticle ≡ 1 mg) and later re-suspended in sterile cell culture media immediately prior to use.

### MicroRNA and Nanoparticle Treatment of NBL Cells in vitro

The Pre-miR™ to miR-34a (30 µM) and the pre-miR-negative control miRNA (negative control 1, Applied Biosystems) were reverse transfected into NB1691, SK-N-AS and HEK293 cell lines using the transfection agent siPORT™ NeoFX™ (Applied Biosystems/Ambion, Austin, TX). For nanoparticle experiments, NB1691, SK-N-AS and HEK293 cell lines were seeded 24 hours prior to treatment and then treated with a final concentration of 6.8×10^9^ particles/ml (40 µg/ml). Each vial of freeze-dried nanoparticles (1mg containing 1.7×10^11^ particles) was resuspended in pre-warmed cell culture media (1 ml) and protected from the light (stock solution of nanoparticles 1.7×10^11^ particles/ml or 1 mg/ml) and appropriate dilutions were made accordingly.

### Fluorescence Analysis

Following treatment with FITC doped nanoparticles, cells were lysed using a RIPA buffer and centrifuged at 14,000 rpm for 15 minutes at 4°C. The relative fluorescent units (RFUs) of isolated supernatant (50 µl) was then detected using a Viktor Microplate luminometer (Molecular Devices, Sunnyvale, CA).

### Acid Phosphatase Assays

Cells were transfected in 96-well plates (1×10^4^ per well). At designated time points post-treatment, cells were washed with PBS and 10 mM *p*-nitrophenol phosphate in sodium acetate (0.1 M) with triton X-100 (0.1%) being added. Plates were incubated at 37°C for two hours in the dark and the reaction was stopped with sodium hydroxide (50 µl 1 M) per well. Absorbance was measured at 405 nm.

### Caspase 3/7 Activity Assay

NB1691, SK-N-AS and HEK293 cells were plated in quadruplicate in 96-well plates. 72 hours after nanoparticle treatment or miRNA transfection, caspase activity was measured using the 3/7 Caspase detection kit from Promega (Madison, WI) in accordance with manufacturers protocols. Luminescence was read on a Viktor Microplate luminometer (Molecular Devices, Sunnyvale, CA).

### In vivo Targeting and Nanoparticle Delivery

All animal experiments were carried out using CB-17/SCID mice (Taconic, Hudson, New York) and were performed in accordance with a protocol approved by the Institutional Animal Care and Use Committee of St Jude Children’s Research Hospital, Memphis, Tennessee. Retroperitoneal tumors were established by injection of 2×10^6^ NB1691^luc^ or SK-N-AS^luc^ cells behind the left adrenal gland via a left subcostal incision under administration of isoflurane (2%), and tumors were allowed to develop over 13 days (n=8 per cohort). Fluorophore-doped-anti-GD_2_-coated NPs, fluorophore-doped-NPs, anti-GD_2_-miR34a-NPs, or anti-GD_2_-miRneg-NPs-treated (10 mg/ml) were administered via lateral tail vein injection to mice with pre-established retroperitoneal neuroblastoma (day 21 for the targeting experiments with FITC doped nanoparticles; day 13 for microRNA encapsulated nanoparticles. Scanning of resected tumours and organs *ex vivo*, or of tumors *in vivo*, was performed using an IVIS Imaging System 100 Series (Xenogen Corporation, Alameda, CA). For experiments using nanoparticles with encapsulated miR-34a or a negative control oligonucleotide, animals in each cohort were tumor size matched using ultrasound and bioluminescent imaging, and each cohort received repeat injections with anti-GD_2_-miRneg-NPs or anti-GD_2_-miR34a-NPs (10 mg/ml) at 3 day intervals (days 14, 17 and 20 post-tumor induction). Prior to imaging, mice received an intraperitoneal injection of D-Luciferin (150-mg/kg, Caliper Life Sciences, Hopkinton, MA).

### Tumor Immunohistochemistry

Formalin-fixed, paraffin-embedded, 4-*µ*m thick tumor sections were stained with rat antimouse CD34 antibody (RAM 34; PharMingen, San Diego, Calif) or KI67 (VP-K451; Vector Laboratories, Burlingame, CA). TUNEL staining was carried out in accordance with previously published protocols [35] and all images were analysed using an Olympus U-SPT microscope as previously described [74].

### Cloning of 3′-UTR of TIMP2 and Luciferase Assays

A 199 bp DNA fragment containing the *TIMP2* 3′UTR was PCR amplified from genomic DNA using flanking primers (Figure S10A) and cloned into the pCR4-TOPO vector (Invitrogen, Grand Island, NY). The DNA fragment of the *TIMP2* 3′UTR was then sub-cloned into psiCHECK2 vector (Promega) to generate psi-*TIMP2*-3′UTR. The resultant clone was sequence verified and used to create the deletion mutant for miR-20b binding sites using the GeneTailor mutagenesis system (Invitrogen). Luciferase assays were performed as previously reported [27]. The sequence of both wild type and mutant 3′ UTR for TIMP2 is presented in Figure S10B.

### Statistical Analysis

The Mann-Whitney non-parametric, one-tailed unpaired test was used to analyze significance of all data. (data is plotted as the median + standard error of the mean).

## Supporting Information

Figure S1 **Evaluation of dye leaching from synthesized silica nanoparticles.**
(PDF)

Figure S2 **NB1691 and SK-N-AS cells were treated with GD_2_-miR34a-NPs in vitro and cell counts were performed.** Relative to GD_2_-miRneg-NP-treated controls, the presence of miR-34a led to a significant reduction in cell numbers in both cell lines 48 and 72 hrs post-treatment (***p<0.001, n=3).
(TIF)

Figure S3 **HEK293 cell sensitivity to miR-34a treatment.** (a) Reverse transfection of HEK293 cells with synthetic premiR-34a resulted in ∼600 fold increase in miR-34a expression levels, relative to miRneg-treated controls, as determined by qPCR (***p<0.001; n=3). (b) HEK293 cells showed a significant reduction in viable cell numbers in the presence of premiR-34a, quantified by acid phosphatase assay (***p<0.001, n=3).
(TIF)

Figure S4 **IMR-90 cell sensitivity to miR-34a treatment.** (a) Human fibroblast (IMR-90) cells which were treated with premiR-34a showed a significant reduction in viable cell number over a 96 hour period using an acid phosphatase assay (***p<0.001, n=3). (b) A corresponding induction of caspase 3/7 activity was noted 72 and 96 hrs post-treatment with premiR-34a (**p<0.01, n=3).
(TIF)

Figure S5 **Tumor size evaluation prior to** ***in vivo*** **nanoparticle treatment.** (a) Ultra-sonography and (b) bioluminescence analysis on day 13 post tumor induction (prior to nanoparticle injection) indicated equivalency of tumor sizes in GD_2_-miR34a-NP versus GD_2_-miRneg-NP treated cohorts.
(TIF)

Figure S6 **Tumor volumes (day 25) and post-mortem tumor weights.** Tumor volumes were measured by ultrasound pre- and post-treatment in NB1691^luc^ and SK-N-AS^luc^ miR-34a treated and control cohorts. Treatment of both neuroblastoma subtypes with GD_2_-miR34a-NPs resulted in a significant reduction in tumor volume (a-b **p<0.01, n=8). Additionally, post-mortem tumor weights were shown to be significantly decreased in GD_2_-miR34a-NP-treated groups relative to their GD_2_-miRneg-NP treated counterparts (c-d **p<0.01, n=8).
(TIF)

Figure S7 **miR-34a profiling in organs subsequent to GD2-miR34a-NP treatment. Q**uantitative PCR analysis of miR-34a expression levels was carried out on liver, kidney and lung tissue from GD_2_-miR34a-NP and control treated cohorts in both SK-N-AS^luc^ (a-c) and NB1691^luc^ (d-f) MiR-34a levels were not significantly increased in healthy tissues subsequent to treatment with GD_2_-miR34a-NP, validating the tumor-specific targeting of the GD_2_-nanaoparticles used in this study.
(TIF)

Figure S8 **Blood chemistry analysis subsequent to GD2-miR34a-NP treatment.** Complete blood chemistries were analysed in response to systemic delivery of GD_2_-miRneg-NP and GD_2_-miR34a-NPs, including serum levels of blood urea nitrogen (BUN, a), alanine aminotransferase (ALT, b), albumin (c), alkaline phosphatase (ALT, d), amylase (e), calcium (f), creatine (g), globulin (h), glucose (i), phosphorus (j), total bilirubin (k) and total protein (l n-=8, mean+sem). Grey-shaded areas indicate guideline ranges as reported by the animal research centre at St. Jude Children’s Research Hospital. Notably, with the exception of creatine levels in NB1691^luc^ and SK-N-AS^luc^ and globulin and total protein levels in SK-N-AS^luc^ treated animals, all values fall within normal ranges; suggesting that administration of the nanoparticles does not appear to adversely affect liver or kidney function within mice in the context of this study.
(TIF)

Figure S9 **Association of low levels of** ***TIMP2*** **mRNA with poor patient overall and event free survival.**
(TIF)

Figure S10 **(A) The sequence of both wild type and mutant 3′ UTR for** ***TIMP2*** **(B)** ***TIMP2*** **3′UTR was PCR amplified from genomic DNA using flanking primers.**
(PDF)

Table S1 **Clinical and biological characteristics of neuroblastoma tumor cohort.**
(XLSX)
