# Supplementary material for: Inhibition of Neuroblastoma Tumor Growth by Targeted Delivery of MicroRNA-34a Using Anti-Disialoganglioside GD2 Coated Nanoparticles
Source: PLoS One. 2012 May 25;7(5):e38129. doi: 10.1371/journal.pone.0038129 (PMC3360657; doi:10.1371/journal.pone.0038129)
Supplement: Figure S1 — Evaluation of dye leaching from synthesized silica nanoparticles. (PDF) [file pone.0038129.s001.pdf]

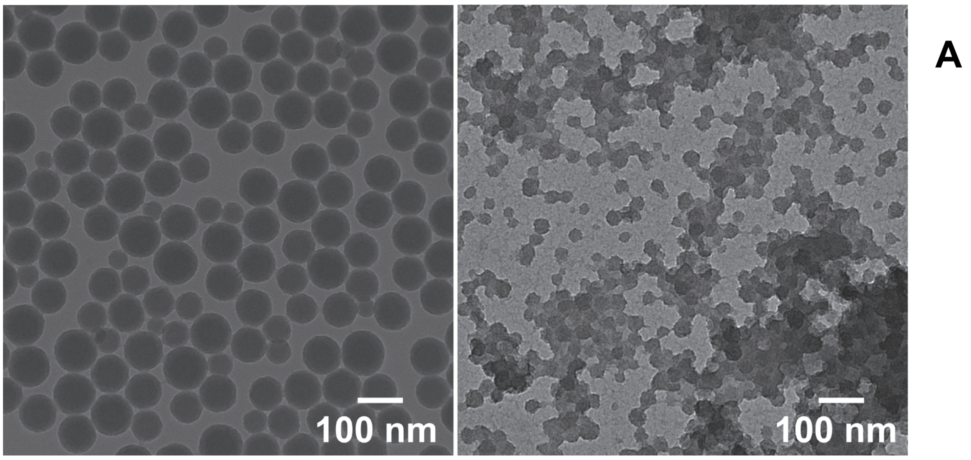

(A) The effects of dissolving the synthesized silica nanoparticles used in this study in phosphate buffer saline (PBS) was evaluated. As shown in transmission electron micrographs, fluorescein dye doped NPs dissolved in ethanol (left) and silica NPs dissolved in phosphate buffered saline [pH = 7.4] (right) were stored for 3 days. The image clearly shows the dissolution of silica nanoparticles in a PBS environment. Park *et al.* noted that the rate of nanoparticle dissolution depended on the size; smaller nanoparticles dissolved faster than large nanoparticles (Park et al., 2008). In our case the nanoparticles appear to dissolve from the external surfaces as opposed to forming a hollow structure. This may be related to the high salt concentration which has a significant effect on the surface potential, charged double layer and colloidal stability of the nanoparticle.

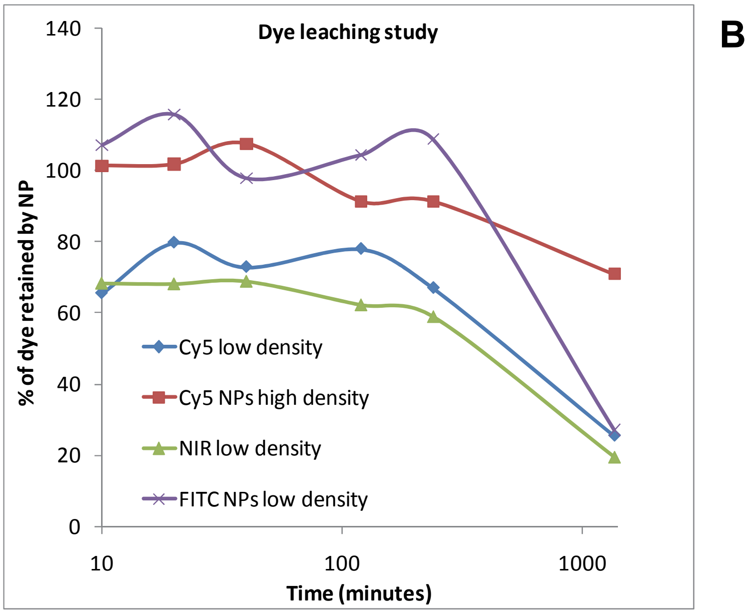

(B) In a further investigation we also studied the rate of dye leaching for microporous and non-porous silica nanoparticle in PBS overnight. Dye leaching occurs rapidly for Cy5, NIR 670 and Fluorescein dye doped nanoparticles in a microporous silica matrix. A drop in signal of approximately 70% was observed overnight. Since the dye is conjugated to the silica matrix, the dye cannot leach via diffusion transport alone, but must also include a hydrolysis step to disassociate from the silica network, therefore confirming a hydrolysis dependent dissolution process.
