# Supplementary figures and images for: Inhibition of Neuroblastoma Tumor Growth by Targeted Delivery of MicroRNA-34a Using Anti-Disialoganglioside GD2 Coated Nanoparticles
Source: PLoS One. 2012 May 25;7(5):e38129. doi: 10.1371/journal.pone.0038129 (PMC3360657; doi:10.1371/journal.pone.0038129)

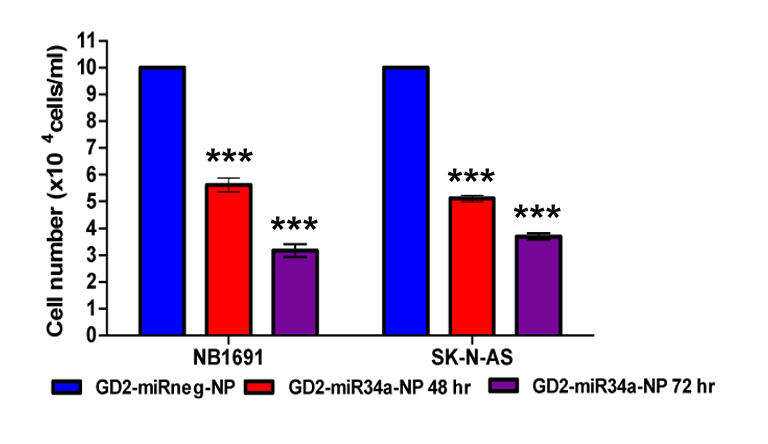

Supplement: Figure S2 — NB1691 and SK-N-AS cells were treated with GD2-miR34a-NPs in vitro and cell counts were performed. Relative to GD2-miRneg-NP-treated controls, the presence of miR-34a led to a significant reduction in cell numbers in both cell lines 48 and 72 hrs post-treatment (***p<0.001, n=3). (TIF) [file pone.0038129.s002.tif]

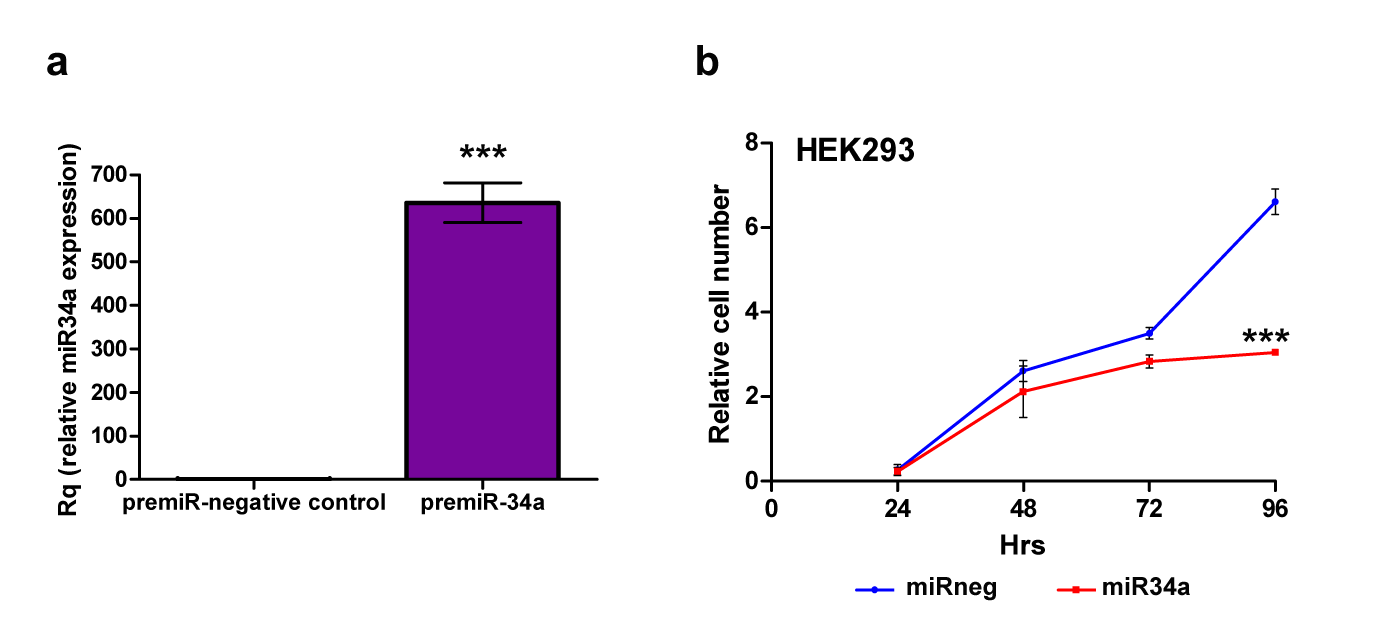

Supplement: Figure S3 — HEK293 cell sensitivity to miR-34a treatment. (a) Reverse transfection of HEK293 cells with synthetic premiR-34a resulted in ∼600 fold increase in miR-34a expression levels, relative to miRneg-treated controls, as determined by qPCR (***p<0.001; n=3). (b) HEK293 cells showed a significant reduction in viable cell numbers in the presence of premiR-34a, quantified by acid phosphatase assay (***p<0.001, n=3). (TIF) [file pone.0038129.s003.tif]

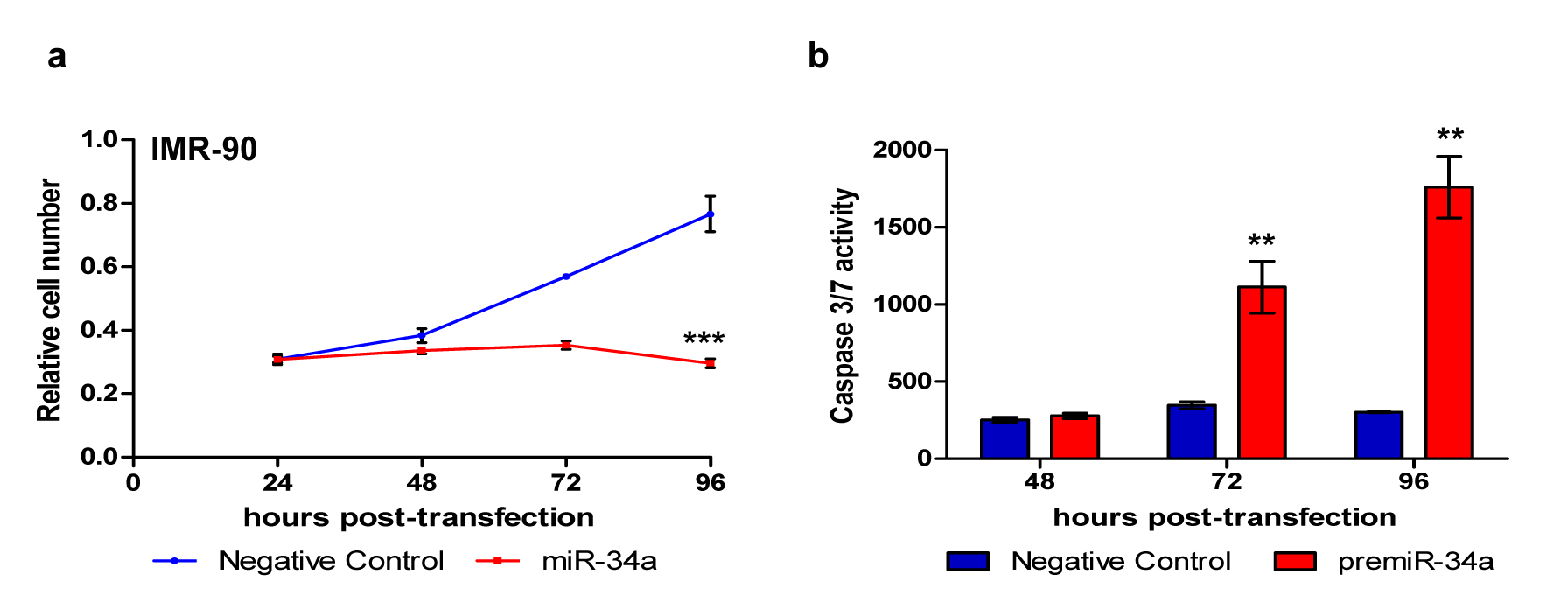

Supplement: Figure S4 — IMR-90 cell sensitivity to miR-34a treatment. (a) Human fibroblast (IMR-90) cells which were treated with premiR-34a showed a significant reduction in viable cell number over a 96 hour period using an acid phosphatase assay (***p<0.001, n=3). (b) A corresponding induction of caspase 3/7 activity was noted 72 and 96 hrs post-treatment with premiR-34a (**p<0.01, n=3). (TIF) [file pone.0038129.s004.tif]

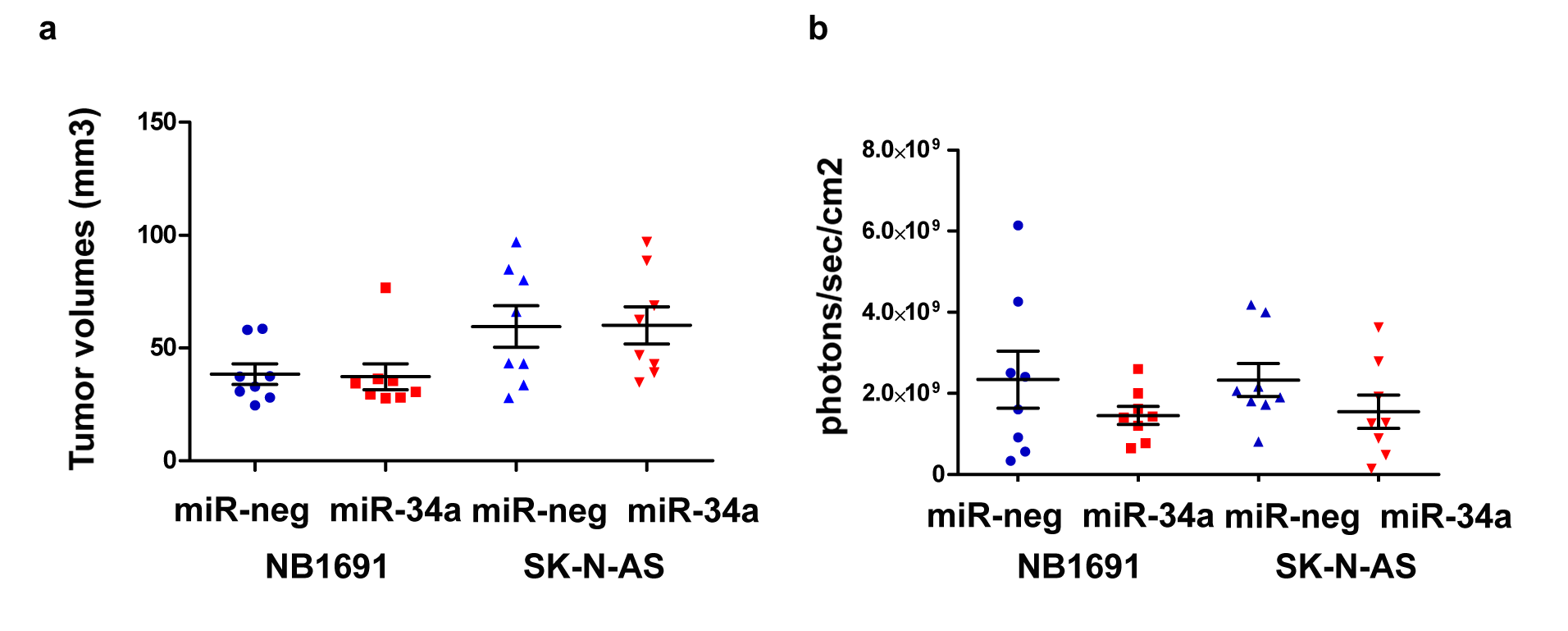

Supplement: Figure S5 — Tumor size evaluation prior to in vivo nanoparticle treatment. (a) Ultra-sonography and (b) bioluminescence analysis on day 13 post tumor induction (prior to nanoparticle injection) indicated equivalency of tumor sizes in GD2-miR34a-NP versus GD2-miRneg-NP treated cohorts. (TIF) [file pone.0038129.s005.tif]

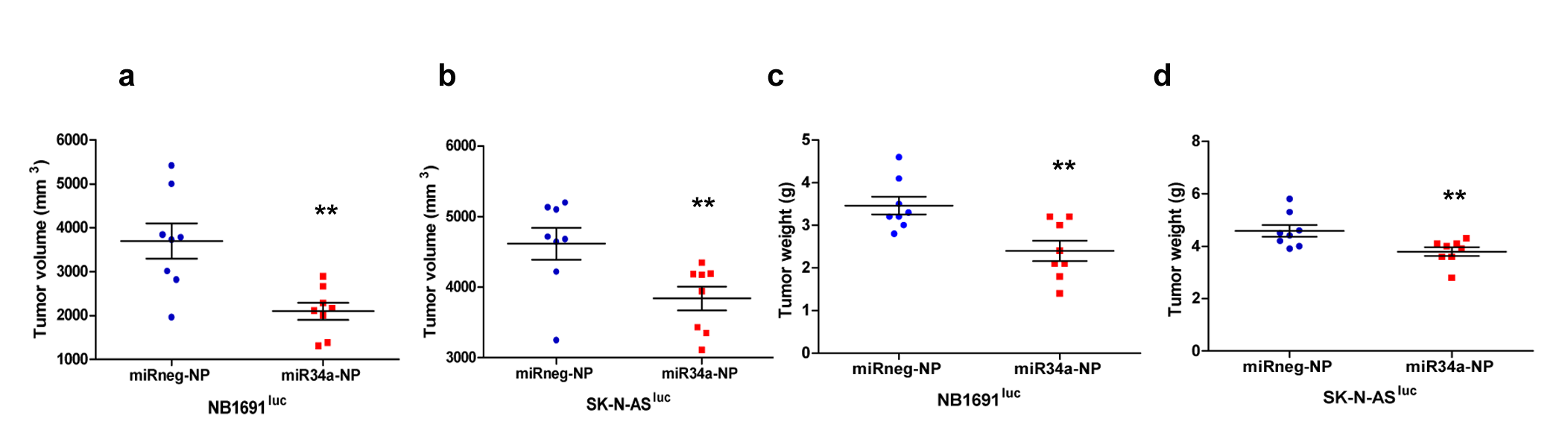

Supplement: Figure S6 — Tumor volumes (day 25) and post-mortem tumor weights. Tumor volumes were measured by ultrasound pre- and post-treatment in NB1691luc and SK-N-ASluc miR-34a treated and control cohorts. Treatment of both neuroblastoma subtypes with GD2-miR34a-NPs resulted in a significant reduction in tumor volume (a-b **p<0.01, n=8). Additionally, post-mortem tumor weights were shown to be significantly decreased in GD2-miR34a-NP-treated groups relative to their GD2-miRneg-NP treated counterparts (c-d **p<0.01, n=8). (TIF) [file pone.0038129.s006.tif]

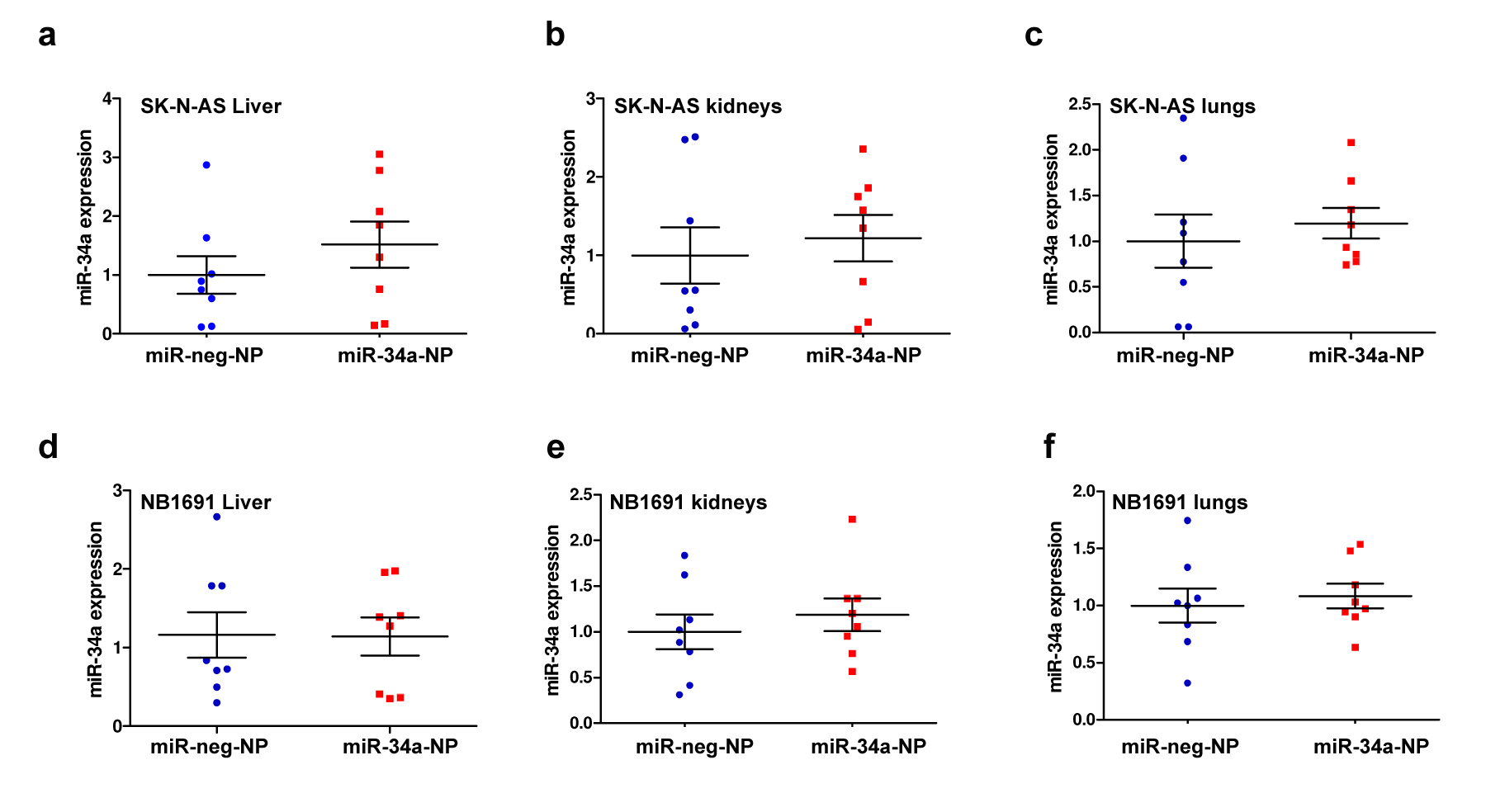

Supplement: Figure S7 — miR-34a profiling in organs subsequent to GD2-miR34a-NP treatment. Quantitative PCR analysis of miR-34a expression levels was carried out on liver, kidney and lung tissue from GD2-miR34a-NP and control treated cohorts in both SK-N-ASluc (a-c) and NB1691luc (d-f) MiR-34a levels were not significantly increased in healthy tissues subsequent to treatment with GD2-miR34a-NP, validating the tumor-specific targeting of the GD2-nanaoparticles used in this study. (TIF) [file pone.0038129.s007.tif]

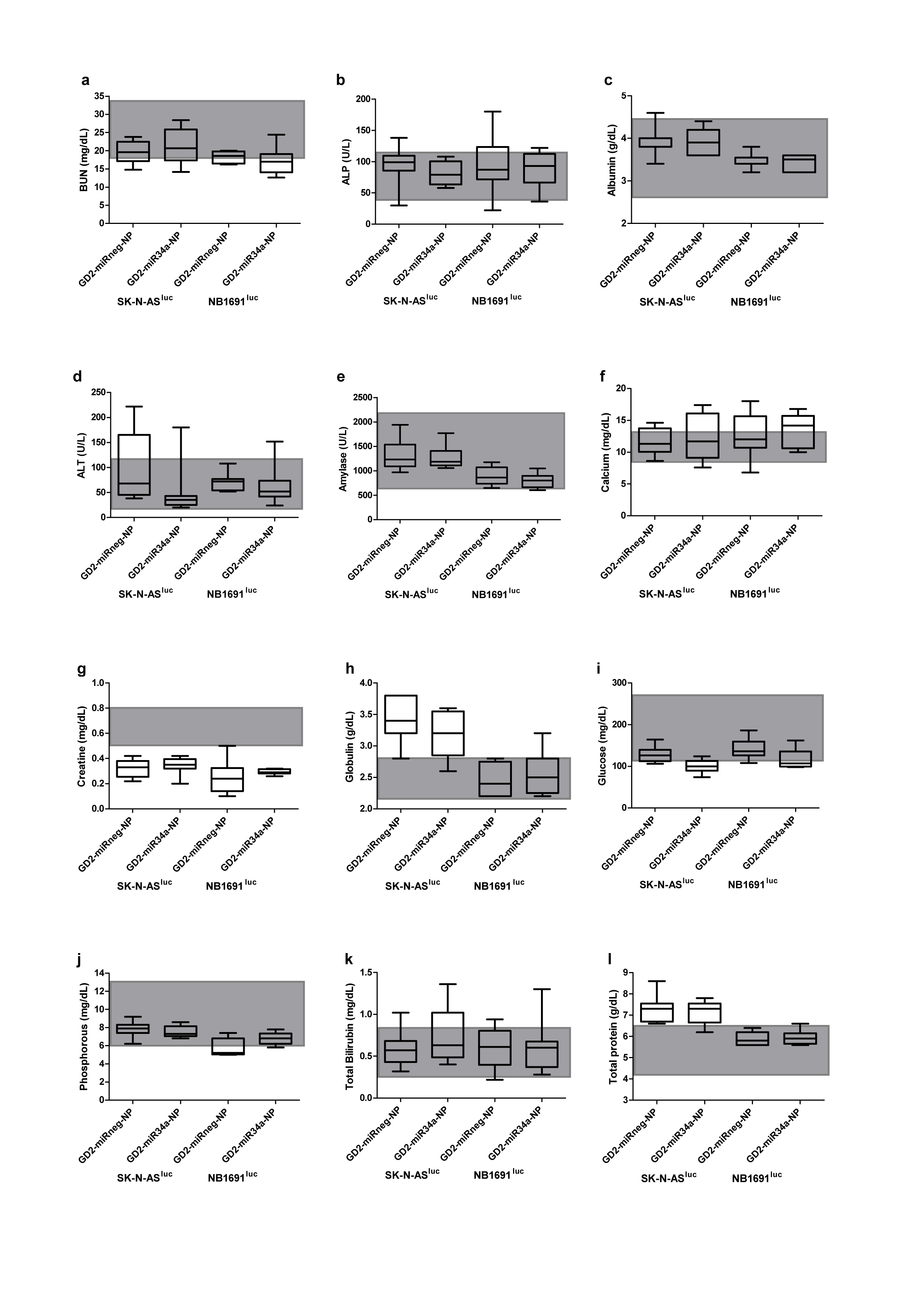

Supplement: Figure S8 — Blood chemistry analysis subsequent to GD2-miR34a-NP treatment. Complete blood chemistries were analysed in response to systemic delivery of GD2-miRneg-NP and GD2-miR34a-NPs, including serum levels of blood urea nitrogen (BUN, a), alanine aminotransferase (ALT, b), albumin (c), alkaline phosphatase (ALT, d), amylase (e), calcium (f), creatine (g), globulin (h), glucose (i), phosphorus (j), total bilirubin (k) and total protein (l n-=8, mean+sem). Grey-shaded areas indicate guideline ranges as reported by the animal research centre at St. Jude Children’s Research Hospital. Notably, with the exception of creatine levels in NB1691luc and SK-N-ASluc and globulin and total protein levels in SK-N-ASluc treated animals, all values fall within normal ranges; suggesting that administration of the nanoparticles does not appear to adversely affect liver or kidney function within mice in the context of this study. (TIF) [file pone.0038129.s008.tif]

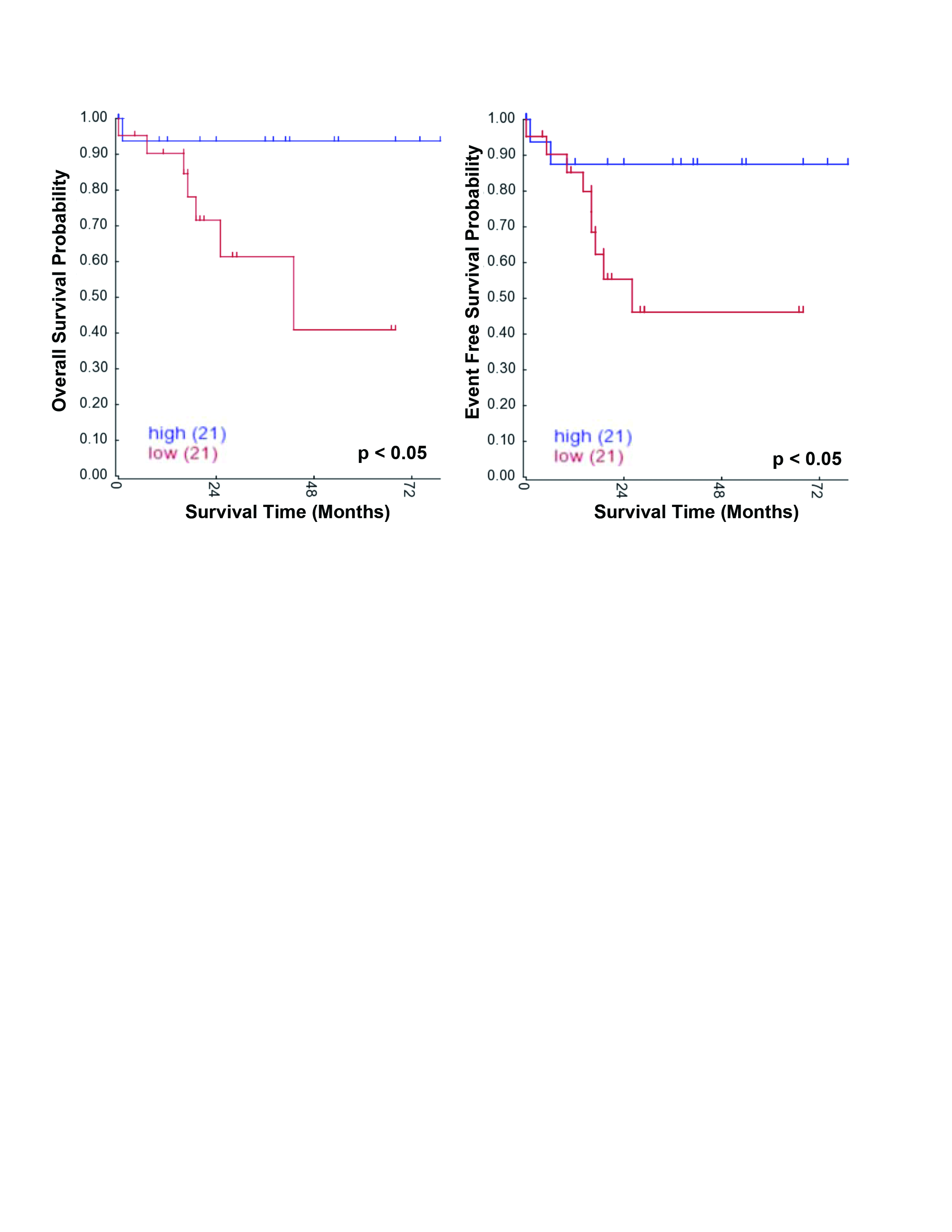

Supplement: Figure S9 — Association of low levels of TIMP2 mRNA with poor patient overall and event free survival. (TIF) [file pone.0038129.s009.tif]
