# Supplementary material for: Inhibition of Neuroblastoma Tumor Growth by Targeted Delivery of MicroRNA-34a Using Anti-Disialoganglioside GD2 Coated Nanoparticles
Source: PLoS One. 2012 May 25;7(5):e38129. doi: 10.1371/journal.pone.0038129 (PMC3360657; doi:10.1371/journal.pone.0038129)
Supplement: Figure S10 — (A) The sequence of both wild type and mutant 3′ UTR for TIMP2 (B) TIMP2 3′UTR was PCR amplified from genomic DNA using flanking primers. (PDF) [file pone.0038129.s010.pdf]

A) Homo sapiens TIMP metallopeptidase inhibitor 2 (TIMP2), mRNA

Sequence source is NCBI Reference Sequence: NM_003255.4

Fragment of *TIMP2* 3’UTR

ATCAGCTGTAATCATTCCTGTGCTGTGTTTTTTATTACCCTTGGTAGGTATTAGACTT**GCACTTTTTT**AAAAA
AAGGTTTCTGCATCGTGGAAGCATTTGACCCAGAGTGGAACGCGTGGCCTATGCAGGTGGATTCCTTCAGGTC
TTTCCTTTGGTTCTTTGAGCATCTTT**GCTTTCATTCGTCTCCCGTCTTTGGTT**

TIMP2 3’UTR with deletion of miR-20b seed site:

CTCGAGATCAGCTGTAATCATTCCTGTGCTGTGTTTTTTATTACCCTTGGTAGGTATTAGACTT_AAAAAAAG
GTTTCTGCATCGTGGAAGCATTTGACCCAGAGTGGAACGCGTGGCCTATGCAGGTGGTTCCTTCAGGTCTTTC
CTTTGGTTCTTTGAGCATCTTTGCTTTCATTCGTCTCCCGTCTTTGGTT

B) Primers used for cloning TIMP2 3’ UTR fragment

oligo name..........	oligo details
**TIMP2-2860-for**	5'-AAAAAGTCGACATCAGCTGTAATCATTCC-3'
**TIMP2-3050-rev**	5'-AAAAAGTTTAAACCAAAGACGGGAGACG-3'
**TIMP2-mut20b-for**	5’-GTAGGTATTAGACTTAAAAAAAGGTTTCTGCATCG-3’
**TIMP2-mut20b-rev**	5’-TGCTGTGTTTTTTATTACCCTTGGTAGGTATTAGAC-3’
